# First review of chronic granulomatous disease in Palestine: clinical and genetic characteristics

**DOI:** 10.3389/fimmu.2026.1764101

**Published:** 2026-03-25

**Authors:** Fatima az-Zahra Thawabteh, Sedrah Abu Rmilah, Aya Siaj, Rawan Abu khdair, Rabee Adwan

**Affiliations:** 1 Medical Research Club, Faculty of Medicine, Al-Quds University, Jerusalem, Palestine; 2 Infectious Diseases Specialist, Al-Makassed Hospital, East-Jerusalem, Palestine; 3 Pediatrics Department, Faculty of Medicine, Al-Quds University, Jerusalem, Palestine

**Keywords:** chronic granulomatous disease, clinical characteristics, genetics, immunodeficiency, lymphadenopathy, Palestine

## Abstract

**Background:**

Chronic granulomatous disease (CGD) is an inborn error of immunity caused by genetic defects in the nicotinamide adenine dinucleotide phosphate oxidase complex, resulting in recurrent severe infections and excessive inflammatory responses. CGD is inherited in X-linked recessive and autosomal recessive patterns. X-linked variants occur in the *CYBB* gene, whereas autosomal recessive variants are found in the *CYBA, NCF1, NCF2, NCF4*, and *CYBC1* genes.

**Results:**

This study analyzed data from 14 CGD patients across 12 families using functional, molecular, and genetic approaches. Among the patients, 11 (78.6%) had autosomal recessive inheritance, and 3 (21.4%) had X-linked recessive inheritance. The most common variants were in the *NCF2* gene, followed by the *CYBB*, *CYBA*, and *NCF1* variants. Consanguinity was observed in 72.7% of the autosomal recessive-CGD families. The most frequent clinical manifestations were lymphadenopathy (71.4%) and gastrointestinal symptoms (64.2%), followed by pulmonary symptoms, organomegaly, and abscesses (57%). The median age at diagnosis was 3 years, with a diagnostic delay ranging from 0 to 4 years, with an average of 17 months.

**Conclusion:**

This study aims to increase awareness among Palestinian healthcare providers and encourage early consideration of CGD in patients with recurrent infections, even in late childhood.

## 1 Introduction

Chronic granulomatous disease (CGD) is a rare, inherited, heterogeneous inborn error of immunity disorder characterized by defects in the genes encoding subunits of the nicotinamide adenine dinucleotide phosphate (NADPH) oxidase complex (1). CGD results from deficits in particular proteins of the phagocyte NADPH oxidase complex, each associated with unique genetic variations. A deficiency in gp91^phox^, encoded by the *CYBB* gene, is the predominant cause of the X-linked recessive (XLR) inheritance pattern. A deficiency in p22^phox^, resulting from variants in the *CYBA* gene, induces an autosomal recessive AR form of the disease. Similarly, variants in the *NCF1* gene result in p47^phox^ deficiency, whereas *NCF2* variants cause a deficiency in p67^phox^; both are inherited in an AR manner. A less common variety of CGD is characterized by p40^phox^ deficiency, resulting from variants in the *NCF4* gene, which is also inherited in an AR manner (2). These genetic variations lead to diminished formation of reactive oxygen species (ROS) by phagocytic cells, which is crucial for the elimination of ingested pathogens (3).

Alongside these classical forms, several rarer genetic etiologies of CGD or CGD-like phenotypes have been identified. Variants in *CYBC1 (*sometimes referred to as EROS), which encodes a chaperone protein essential for stabilizing the gp91^phox^–p22^phox^ heterodimer, result in a unique AR form currently identified as CGD type 5 (4). Variants in *RAC2*, a hematopoietic-specific GTPase involved in both NADPH oxidase activation and neutrophil chemotaxis, have also been linked to CGD-like immunodeficiency characterized by recurrent infections and impaired ROS generation. Additionally, in rare cases, severe *G6PD* deficiency can compromise NADPH availability, mimicking the oxidative burst defect observed in CGD (5). Other genes, such as *MPO* and *GSS*, are associated with CGD-like presentations through indirect disruption of ROS-mediated microbial killing (6). Including these atypical genetic variants in the diagnostic workup enhances the understanding of CGD genetic heterogeneity and supports more accurate diagnosis and tailored clinical management.

Consequently, patients with CGD exhibit a markedly diminished ability to eliminate specific bacteria and fungi, rendering them susceptible to recurring, potentially fatal infections. Patients with CGD often form tissue granulomas as a result of chronic inflammation, leading to significant tissue damage and disruption of normal organ function. The clinical manifestations of CGD can vary on the basis of particular genetic variations. However, the hallmark of this disease is susceptibility to infections caused by catalase-positive bacteria, fungi, and mycobacterial species, which are less efficiently eliminated by the immune system (1, 7).

The clinical spectrum of CGD ranges from mild to severe in both the XLR and AR forms, with the AR subtype typically exhibiting milder manifestations (3). The incidence of CGD is estimated to affect approximately 1 in 200,000 live births in the United States. The highest frequency is anticipated to be 1.5 per 100,000 in Palestinian populations and 1.05 per 100,000 in Jewish communities in occupied Palestine (1).

The diagnosis of CGD involves the quantification of ROS, including superoxide (O_2_^−^) and hydrogen peroxide (H_2_O_2_), generated by the NADPH oxidase complex in phagocytes derived from peripheral blood. This is usually performed after the *in vitro* activation of phagocytes with drugs such as phorbol-12-myristate-13-acetate (PMA) or Toll-like receptor 4 (TLR4) ligands, including *Escherichia coli* (*E. coli*) or lipopolysaccharides (LPS). These stimuli trigger an oxidative burst, facilitating the evaluation of ROS generation and NADPH oxidase activity, which are essential for confirming CGD. Superoxide (O_2_^−^) can be measured using chemiluminescence or the conventional nitroblue tetrazolium (NBT) assay, which is commonly employed in CGD diagnoses. Hydrogen peroxide (H_2_O_2_) is often quantified using the flow cytometry-based dihydrorhodamine (DHR) assay, which is renowned for its remarkable sensitivity and is considered the gold standard for CGD; this method involves evaluating the functionality of the NADPH oxidase complex (8). However, both medications and nonspecific ROS production can contribute to false-positive results in the DHR assay for CGD (9). Medications such as acetaminophen, metamizole, and mesalazine interfere with ROS production, leading to misleading fluorescence readings that may falsely suggest a normal or enhanced respiratory burst. Additionally, nonphagocytic cells or nonspecific sources of ROS in the testing environment can also skew results, further complicating the interpretation of DHR-based assays (10). Accordingly, genetic testing provides definitive confirmation of CGD by identifying pathogenic variants in the genes encoding components of the NADPH oxidase complex, including *CYBB, CYBA, NCF1, NCF2*, and *NCF4*. Identification of a causative genetic mutation is therefore considered the gold standard for the diagnosis of CGD (11).

The typical age at which CGD is diagnosed is between 2.7 and 3.0 years (2). Globally, approximately fifty percent of newly diagnosed primary immunodeficiency (PID) patients are adults aged 25 years and older. The rarity and limited awareness of this disease among medical practitioners frequently results in delayed diagnoses (2).

CGD exhibits a highly varied phenotype, marked by infectious or inflammatory consequences that can affect the skin, soft tissues, gastrointestinal tract, lungs, central nervous system, and other organs. The extensive variety of presentations renders CGD an illness that any physician might encounter (2).

This study aims to characterize Palestinian CGD patients. This study outlines the disease burden and specifies the demographic, clinical, laboratory, radiological, and genetic characteristics of CGD patients. It additionally compares these findings with those from other countries, highlighting both similarities and discrepancies. By initiating appropriate diagnostic testing and management strategies, we aim to improve the quality of life of patients and prevent future complications. This study aims to provide practical recommendations to prevent misdiagnosis and facilitate prompt treatment.

## 2 Methodology

### 2.1 Research design

A retrospective chart review was conducted on patients with a confirmed diagnosis of CGD. The study was ethically approved by both the hospital and the Ethics Committee at Al-Quds University.

### 2.2 Study population and sample

The study included all patients diagnosed with CGD at any age who received care at Al-Makassed Hospital, the main tertiary referral center for CGD in Palestine, between 2015 and 2024. Only patients with genetically confirmed diagnoses were included. Data were collected on demographic characteristics (sex, age at diagnosis, and parental consanguinity), clinical and laboratory findings, genetic variants, treatment modalities, and survival outcomes. Patients diagnosed solely by DHR testing without genetic confirmation were excluded.

### 2.3 Genetic testing and dihydrorhodamine test

Sequencing of the patient’s phagocyte oxidase (phox) genes is also performed to determine the exact molecular defect. Both medications and nonspecific ROS production can contribute to false-positive results in the DHR assay for CGD (9).

Genomic analysis was performed by whole-exome sequencing using the IDT xGen Exome Research Panel v2.0 combined with the xGen Human mtDNA Research Panel v1.0, followed by high-throughput sequencing on the Illumina NovaSeq 6000 platform. FASTQ files were generated and processed using the Geneyx platform, with secondary analysis conducted via the DRAGEN pipeline aligned to the hg19 human reference genome. Clinically relevant mitochondrial DNA (mtDNA) variants were reported when heteroplasmy levels exceeded 10%. For trio-based exome analysis, uniparental disomy (UPD) was assessed via the UPDio tool.

### 2.4 Data collection procedures

A total of 14 patients were diagnosed with CGD. The patients’ medical history included their date of birth and sex, along with details about any family-related cases of CGD, including the presence of consanguinity between parents. The mode of transmission, whether XL or AR, and the specific genetic variant identified through genetic diagnosis were also documented. The key timelines included the date of first symptoms, the official diagnosis, and the diagnostic methods used, such as genetic testing, nitroblue tetrazolium (NBT), or immunomodulatory assays, along with their results. Information on antimicrobial prophylaxis, including the type, was also collected. Whether the patient underwent hematopoietic stem cell transplantation (HSCT) was documented, along with the cause of death if applicable.

A standardized data collection sheet, developed from previous literature and in consultation with clinical experts, was used to gather demographic, clinical, medical laboratory, radiological, and genetic details on patients with CGD during the study period. The data were anonymized, and statistical analysis was performed using IBM SPSS for Windows, Version 23.0. Continuous variables are presented as medians with interquartile ranges, and categorical variables are presented as frequency ranges and ratios.

## 3 Results

### 3.1 Patients’ characteristics

This study enrolled a total of 14 patients with a confirmed diagnosis of CGD from 12 unrelated families, including 4 females and 10 males (**Table 1**). All patients met the diagnostic criteria for CGD on the basis of a combination of clinical features, dihydrorhodamine 123 (DHR) and nitroblue tetrazolium (NBT) assays, and the identification of pathogenic variants in genes encoding the NADPH oxidase complex. Among these patients, 13 patients (92.86%) underwent DHR testing, whereas only 1 patient (7.1%) underwent NBT testing. A notable family history was observed in four patients. Patients’ residences were from various governorates of the West Bank.

**Table 1:** General characteristics of the 14 chronic granulomatous disease patients: M-month-old, AR autosomal recessive, XLR X-linked recessive, NA not applicable.

Patient number	Gender	Age at presentation (years)	Age at diagnosis (years)	Alive/Dead	Family history	Parents consanguinity	Type of inhabitancy	Mutation
P1	Female	10	12	Alive	Sister w/CGD	2^nd^ degree	AR	*CYBA* c.164C>G (p.Pro55Arg9)
P 2	Male	2M	2M	Alive	Free	3^rd^ degree	AR	NCF2 c.1171_1175delAAGCT (p.Lys391GlufsTer9)
P3	Male	7M	1	Alive	Free	3^rd^ degree	AR	*NCF2* c.1171_1175delAAGCT (p.Lys391GlufsTer9)
P4	Male	2M	3M	Alive	Free	3^rd^ degree	AR	NCF2 c.1171_1175delAAGCT (((p.Lys391GlufsTer9)
P5	Female	4M	2	Alive	Brother w/CGD	3^rd^ degree	AR	NCF2 c.1171_1175delAAGCT T (p.Lys391GlufsTe (p.Lys391GlufsTer9)
P6	Male	6M	4	Alive	Free	No	XLR	CYBB c.1226C>A p.Ala409Glu
P7	Male	14	17	Alive	Free	No	XLR	CYBB c.433G>A (p.G145R)
P8	Female	2M	4	Dead	Sister w/CGD	2^nd^ degree	AR	CYBA c.164C>G (p.Pro55Arg)
P9	Female	6M	8	Alive	Free	2^nd^ degree	AR	NCF2 c.1171_1175delAAGCT p.Lys391GlufsTer9
P10	Male	5	5	Dead	Free	No	AR	NCF2 c.1263C>A (p.Cys421Ter)
P11	Male	2M	3M	Alive	positive	No	XLR	CYBB (c.483 + 1G~A) p.Ala113GlufsX3
P12	Male	10M	1	Alive	Sister w/CGD	No	AR	NCF2 c.1171_1175delAAGCT (p.Lys391GlufsTer9)
P13	Male	12	15	Alive	Free	No	AR	NCF2 c.1171_1175delAAGCT (p.Lys391GlufsTer9)
P14	Male	9	11	Alive	Free	2^nd^ degree	AR	NCF1 c.75_76delGT p.Val26LeufsTer

The median age of disease onset was 8.5 months (range: 0–14 years), and the median age at diagnosis was 3 years (range: 0–17 years), resulting in a mean diagnostic delay of 17 months. This excluded patient 10, whose exact age at presentation was unavailable.

### 3.2 Neutrophil function and genetic studies

**Figure 1:**
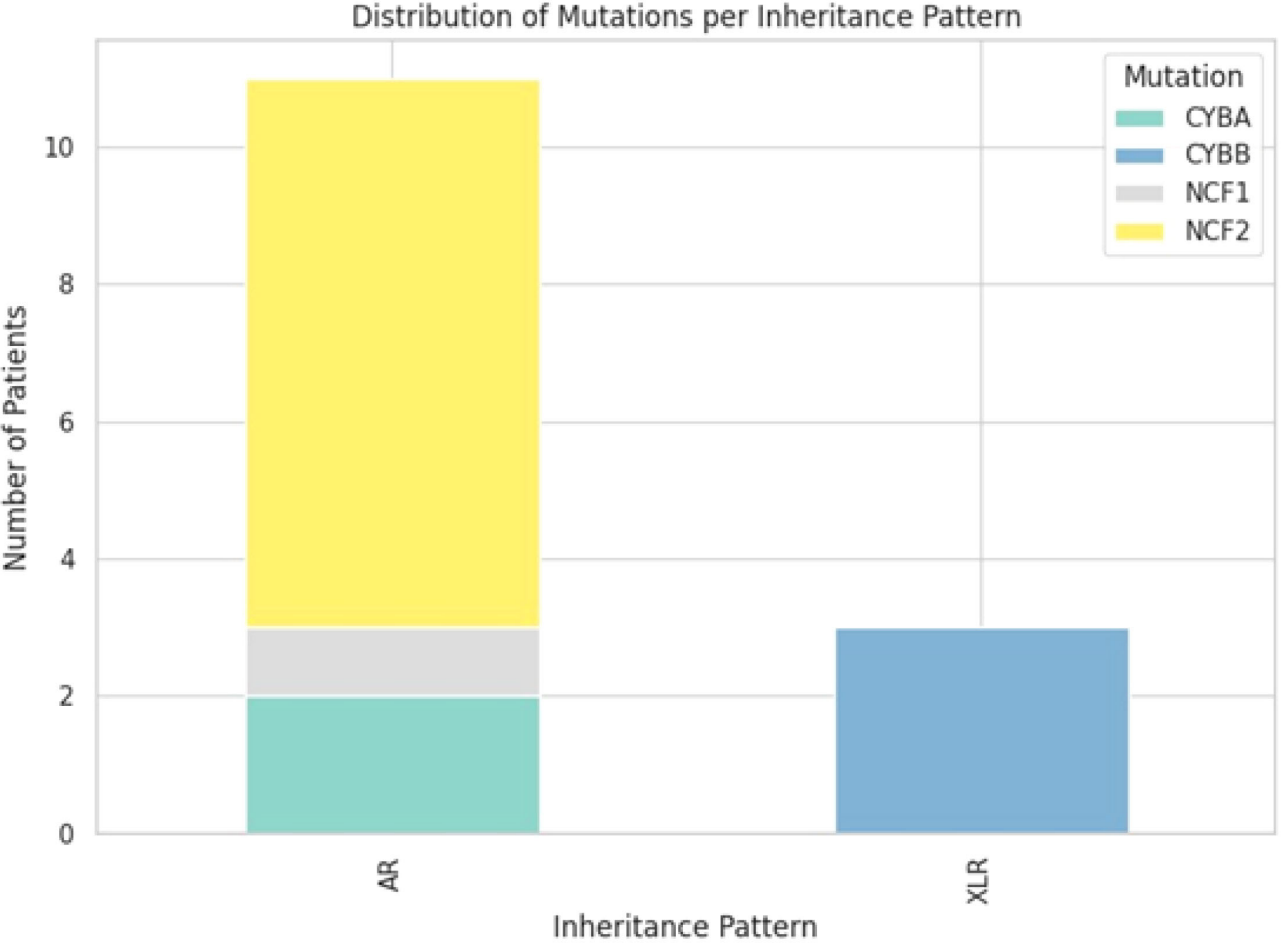
Prevalence of genetic patterns and variants of CGD inheritance among Palestinian patients.

Genetic analysis revealed that 78.6% of patients had AR CGD, whereas 21.4% had XLR CGD. The most commonly involved gene was AR, with a p67^phox^ variant encoded by *NCF2* identified in 8 patients, followed by XLR, with a gp91^phox^ variant encoded by *CYBB* in 3 patients; AR, with a p22^phox^ variant encoded by *CYBA* in 2 patients; and AR, with a p47^phox^ variant encoded by *NCF1* in 1 patient. No patients had variants in p40^phox^ (encoded by *NCF4*) or EROS (encoded by *CYBC1*) (**Figure 1**). An identical frameshift mutation in the NCF2 gene, c.1171_1175delAAGCT (p.Lys391GlufsTer9), was identified in seven patients (P2, P3, P4, P5, P9, P12, and P13) from 6 unrelated families. Siblings 1 and 8 carried a CYBA variant, while siblings 5 and 12 carried an NCF2 variant. The prevalence of XLR and AR forms differed significantly, highlighting the high rate of AR inheritance in Palestine, which is associated with a high rate of consanguinity. In our study, 72.7% of patients had consanguineous parents: 4 patients had second-degree relatives, and another 4 had third-degree relatives.

### 3.3 Clinical manifestations

**Figure 2:**
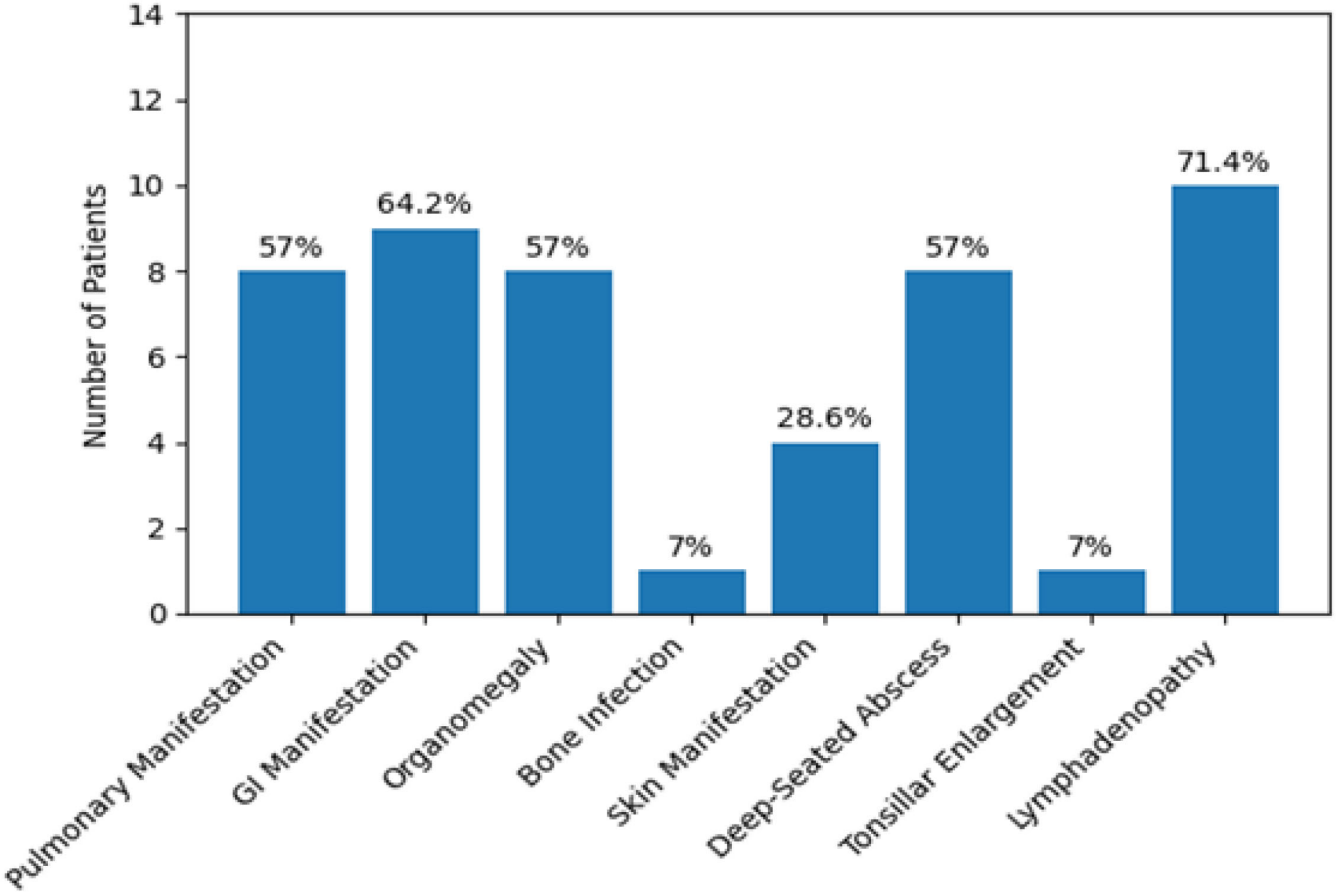
Frequencies and percentages of clinical manifestations observed in CGD patients.

The most common clinical presentation was lymphadenopathy, observed in 10 patients (71.4%; **Figure 2**). Among these, 4 patients (28.6%) presented with lymphadenitis, while the remaining 6 patients (42.8%) exhibited only lymphadenopathy. Lymph node enlargement was predominantly cervical (n = 2). Other affected sites included diffuse, subauricular, inguinal, para-aortic, iliac, axillary, hilar, retroperitoneal, and postauricular regions. The remaining documented clinical manifestations were categorized as infectious or non-infectious, as detailed below.

Non-infectious manifestations included gastrointestinal (GI) involvement and organomegaly. GI manifestations were noted in 9 patients (64.2%) and comprised diarrhea (n = 5, 35.7%), abdominal pain (n = 3, 21.4%), abdominal distention (n = 3, 21.4%), vomiting (n = 1, 7.1%), and poor weight gain (n = 1, 7.1%). Organomegaly was identified in 8 patients (57%), including hepatomegaly in 6 patients (42.9%), splenomegaly in 1 patient (7.1%), and hepatosplenomegaly in 1 patient (7.1%). Additional non-infectious manifestations included failure to thrive, primary amenorrhea, bilateral nephrolithiasis, and complex neurological features.

Infectious manifestations involved multiple organ systems. Pulmonary infections were observed in 8 patients, with recurrent chest infections being the most frequent presentation (n = 7, 50%). Lung empyema and pulmonary infiltrates were each identified in 1 patient (7.1%). Deep-seated abscesses occurred in various anatomical locations, including the perianal region (n = 2, 14.3%) and the liver (n = 2, 14.3%), while isolated abscesses were also noted in the crypts, cheeks, eyes, brain, dental regions, and vaccine injection sites, each in 1 patient (7.1%). Osteomyelitis was documented in Patient 1 (right iliac bone), and recurrent urinary tract infections were reported in Patient 8. Otitis media was recorded in Patient 9, who also experienced recurrent enterovirus infections presenting as severe, prolonged hand, foot, and mouth disease.

Additional clinical features included skin and soft tissue manifestations. Skin ulcers were identified in Patient 2, skin rash in Patient 3, and both skin manifestations in Patient 9. Patient 8 had developed a tonic clonic seizure, an urgent brain CT done which revealed a 2.5-cm intra-axial mass in the right frontal lobe associated with grade 1 vasogenic edema. Following frontal craniectomy, the lesion was identified as an abscess, which was surgically evacuated; culture of the abscess fluid grew *Pseudomonas* spp. and *Acinetobacter* spp.

In terms of inflammatory/autoimmune manifestations, systemic lupus erythematosus, granulomatous dermatitis, and inflammatory bowel disease were encountered in 3 patients (21.4%). Patient 8, who had undergone splenectomy, developed a lupus-like syndrome in addition to complex generalized tonic–clonic seizures. Patient 14 was diagnosed with granulomatous dermatitis via a skin biopsy. Patient 1 had a clinical presentation resembling severe inflammatory bowel disease (IBD), with severe transmural intestinal inflammation.

Regarding infective microorganisms, patient 1 underwent bronchoscopy, which identified *Aspergillus* spp. by staining, and granulomas were observed via histopathology. *Staphylococcus aureus* was detected in a liver abscess from Patient 7. *Mycobacterium tuberculosis* was detected in Patient 2, and enterovirus was detected in Patient 9. Pseudomonas spp. and Acinetobacter spp. were detected in the brain abscess in Patient 8. However, cultures from the majority of the other patients revealed no bacterial growth, as they were referred to out-of-care settings while on broad-spectrum antibiotics.

Other radiological and pathological findings observed in patients are summarized in **Tables 2**, **3**, respectively.

**Table 2:** Radiological findings of patients with CGD.

System	Scan Type	Patient	Findings
Respiratory	Chest CT	P1	Ground glass opacity
	Chest CT	P2	5.5 * 4 cm cavitary lesion with an air–fluid level in the right upper lobe
	Chest CT	P3	Consolidation with airbronchgram, with atelectatic changes seen in the left upper lobe
	Chest CT	P6	Few small focal patchy ground-glass opacities and consolidative areas, associated with mild interstitial septal thickening and mild bronchial wall thickening (more pronounced in the lower lobes), in addition to a few small subcentimeter mediastinal lymph nodes
	Chest X-ray	P10	Left lung infiltration due to pneumonia
	Chest CT	P13	Right-sided empyema
Hepatobiliary/GI	Abdomen & Pelvic US	P1	Ill-defined, macrolobulated heterogeneous collection measuring approximately 4 * 3 cm, with features suggestive of a liver abscess.
	Whole Body CT	P2	Hepatic, splenic, and renal hypodense lesions, suggestive of a disseminated malignant metastatic process
	Abdomen CT	P13	Hepatosplenomegaly, multiple enhancing masses in the right hepatic lobe, and multiple enlarged para-aortic lymph nodes
Lymph Nodes	CT with Contrast	P5	Small mesenteric lymph nodes
	CT with Contrast	P9	Multiple cervical lymph nodes, the largest measuring 4 mm; multiple axillary lymph nodes, the largest on the left measuring 9 mm; and bilateral inguinal lymph nodes, the largest measuring 1 cm
Neurological	Brain CT	P8	2.5 cm right frontal lobe intra-axial mass with grade 1 vasogenic edema
Musculoskeletal	Pelvic X-ray	P1	3 * 4 cm osteolytic lesion within the cortex, located in the most lateral aspect of the right iliac bone

**Table 3:** Pathological findings of patients with CGD.

System	Biopsy site	Patient	Pathologic features
Gastrointestinal	Colon	P1	Inflammation, cryptitis, crypt abscesses, and epithelioid granulomas
	Rectosigmoid Colon	P4	Mild acute inflammation/colitis
Respiratory	Bronchial Biopsy	P1	Granulomatous inflammation with suppuration
	Bronchoalveolar Lavage	P1	Reactive inflammation composed of approximately 80% macrophages and multinucleated giant cells
	CT-Guided Lung Biopsy	P2	Giant cells, lymphocytes, and extensive foci of necrosis
	Lung Biopsy	P14	Eosinophilic pneumonia
Liver	Right Liver Lobe Biopsy	P7	Features of CGD include portal tract inflammation and interface hepatitis
Skin	Skin Biopsy	P14	Granulomatous dermatitis

### 3.4 Management and outcomes

Prophylactic trimethoprim-sulfamethoxazole (cotrimoxazole) and itraconazole were initiated in all 14 patients at the age of diagnosis. Additionally, immunomodulatory therapy was utilized to manage autoinflammation and immune dysregulation. Patient 1, in particular, underwent biological treatment with STELARA (ustekinumab) at age 13.

Five patients (35.7%) underwent bone marrow transplantation outside Palestine. 2 patients died: patient 8 at the age of 23 due to complications from chickenpox, and patient 10 due to an unknown cause at the age of 9 years. Follow-up data were available for all patients. At the time of publication of this review, the other 12 patients were alive. The overall survival rate is 85.7%.

## 4 Discussion

Chronic granulomatous disease (CGD) is a rare disorder with varied prevalence across diverse ethnic populations. The projected worldwide incidence is approximately 1 in 200,000 live births (12). However, in Palestine, a small nation with a significant prevalence of consanguineous marriages (approximately 40% of unions), there is no published information concerning the occurrence of CGD (13).

In the present study, the majority of patients (78.6%) presented with AR CGD, with genetic variations distributed as follows: *NCF2, CYBA, and NCF1*. The notable prevalence of the AR form compared with the XL form in this study is associated with the elevated rate of consanguinity (57.14%) in Palestine. These findings correspond with analogous studies from the Middle East, where consanguineous marriages are prevalent, including nations such as Egypt, Iran, and Israel (7, 14, 15). In contrast, the XL variant of CGD is more common in Western nations, including the United States, Europe, Mexico, and Japan (10, 16, 17).

Despite the significant prevalence of AR CGD, 71.4% of our patients were male. This differs from the global male–female ratio for AR inheritance, which generally shows no substantial variation (48–57%). This discrepancy may be attributed to the limited sample size in our study.

X-linked chronic granulomatous disease (XL-CGD) typically manifests at an earlier age with more severe symptoms than the autosomal recessive type. 3 Considering the prevalence of the AR type in our cohort, the median age of disease onset (8.5 months) and diagnosis (3 years) was lower than that documented in the majority of studies, including those from Europe, Latin America, Turkey, the UAE, and Iran (3, 15, 18, 19). However, they surpassed the records in India and China, where routine BCG immunization at birth correlates with early presentation and diagnosis (20, 21). The BCG vaccine is routinely administered to newborns in Palestine, and all of our patients received it. Nonetheless, our data indicated the absence of BCG-related adverse effects. This may be due to caregivers being oblivious to the symptoms their child exhibited postvaccination, or they may have perceived it as a benign vaccine reaction, similar to reactions from other immunizations. Conversely, families may not have associated the child’s history of BCGitis with CGD.

The interval between symptom onset and diagnosis varied from 0 to 4 years, with a mean delay of 17 months following initial presentation. The delay is due to insufficient facilities and funding for basic immunodeficiency treatment. For example, genetic analysis is unavailable locally, necessitating the shipment of materials overseas for testing.

Lymphadenopathy was the predominant clinical manifestation observed in our patients, followed by chronic diarrhea. These findings correspond with those documented in the Hacettepe experience, where lymphadenopathy was similarly the predominant clinical manifestation (22). Conversely, research from different areas, including Latin America, Egypt, and Mexico, has indicated divergent outcomes, with pneumonia emerging as the predominant clinical manifestation (10, 14, 18). Additional features included pulmonary manifestations, organomegaly, abscesses, dermatological manifestations, bone infections, and tonsillar enlargement, indicating that CGD is linked to a diverse array of clinical characteristics.

Like other primary immunodeficiencies, the clinical challenge in CGD encompasses not only immunological deficiency but also a dysregulated inflammatory response (8). This was particularly evident in the gastrointestinal manifestations, prominently featuring persistent diarrhea as the predominant symptom, followed by stomach pain, abdominal distention, vomiting, and inadequate weight gain. Comparable results were documented in Latin America and at Hacettepe, where manifestations of colitis, such as diarrhea, abdominal pain, and rectal discomfort, were noted (22). However, similar symptoms were documented with greater frequency in studies conducted in Europe and North America, perhaps owing to the prevalence of XL-CGD in these areas.

The pulmonary symptoms identified in this study align with studies from India, where recurrent chest infections were the most common, followed by lung empyema and pulmonary infiltrates. This differs from the Hacettepe experience, which indicated a distinct pattern, with lymphadenopathy (mostly mediastinal and hilar) and nodules as the predominant lung findings (22). These disparities underscore the heterogeneity in pulmonary symptoms of CGD among diverse populations.

In patients with CGD, noninfectious complications, including gastrointestinal, pulmonary, and rheumatological manifestations, may arise due to chronic and uncontrolled inflammation and recurrent infections. Inflammatory complications may occasionally obscure infectious signs, resulting in diagnostic delays. This pattern was apparent in a patient with a documented history of systemic lupus erythematosus (SLE), underscoring the shared characteristics between chronic granulomatous disease (CGD) and autoimmune or autoinflammatory disorders. Macrophage activation syndrome (MAS), a rare yet life-threatening hyperinflammatory condition, has been documented in patients with chronic granulomatous disease (CGD). MAS is defined by the uncontrolled activation of macrophages and T cells, resulting in fever, cytopenias, hyperferritinemia, and multiorgan dysfunction (23). Timely identification and intervention with corticosteroids, intravenous immunoglobulin (IVIG), cyclosporine, or HLH-direct protocols are essential for enhancing results (23). These findings highlight the necessity of including CGD in the differential diagnosis of children exhibiting early-onset or atypical inflammatory conditions. Pediatricians and gastroenterologists must be especially attentive in identifying these manifestations and performing suitable diagnostic assessments for CGD in these instances.

This analysis identified *Aspergillus*, *Mycobacterium tuberculosis*, and *Staphylococcus aureus* as documented causal agents, all of which are catalase positive, as noted in the literature. Nonetheless, cultures from the majority of patients did not produce bacterial growth. The stagnation in growth may be ascribed to the widespread administration of antibiotics for both treatment and prophylaxis. As Al-Makassed Hospital is a tertiary care facility, numerous patients initiate broad-spectrum antibiotics prior to receiving medical consultation or being admitted to the hospital, which likely inhibits the growth of bacteria in the cultures. Therefore, it is essential to obtain humoral and tissue cultures before initiating treatment in patients with CGD to ascertain the etiological agent and inform suitable therapeutic strategies.

Upon diagnosis, all patients with CGD should be administered prophylactic therapy comprising both antibacterial and antifungal drugs. Trimethoprim-sulfamethoxazole (cotrimoxazole) is the preferred antibacterial prophylaxis because of its substantial reduction in bacterial infection incidence and cost-effectiveness. Itraconazole is the predominant antifungal agent employed for the prevention of invasive Aspergillus and other mold diseases. Nevertheless, posaconazole may be regarded as an option on the basis of regional antifungal resistance patterns and individual patient variables. Immunomodulatory therapy may be necessary to manage autoinflammation and immunological dysregulation, but allogeneic HSCT offers a potential cure (8).

The routine administration of interferon-gamma for prophylaxis remains contentious. It is more prevalent in the USA than in Europe (24). A multicenter trial on interferon-gamma in patients with CGD demonstrated a 77% reduction in serious infections relative to the placebo group, with a more pronounced impact observed in younger patients when it was used in conjunction with antibiotic prophylaxis (23). In Palestine, interferon-gamma is not commonly administered to CGD patients owing to its restricted availability and high cost.

All 14 patients received medical therapy, and five of them (35.7%) underwent bone marrow transplantation. The incidence of HSCT in our study was inferior to that documented in the literature, primarily attributable to factors including patients’ and families’ inclination toward traditional follow-up, challenges in identifying a matched donor, the emergence of end-organ damage, constraints in executing the procedure in Palestine, and the necessity for expanded referral alternatives.

The mortality rate in this study was 2 patients (14.28%), both of whom had the AR form of CGD, with one having undergone HSCT. This rate is relatively low compared with that reported in the literature, possibly attributable to the predominance of the AR form, which is typically less severe, as well as the small sample size. Initial research on CGD indicated a mortality rate of more than 50% by age 6 and approximately 65% by age 7.25. However, advancements in medical care have markedly reduced mortality rates. A recent study revealed that 50% of CGD patients exhibited a median survival of 25 years post-diagnosis. The median life expectancy for CGD patients in Europe is notably high—37.8 years for XL-CGD patients and 49.6 years for AR-CGD patients. The overall survival rate for CGD is 90%, with numerous patients reaching adulthood, but other studies have reported long-term survival rates of 81%. However, survival rates are generally lower in underdeveloped nations (21).

CGD is a rare condition that should be considered in the differential diagnosis of chronic inflammatory and infectious diseases. Although CGD is generally detected in early childhood, typically within the first 1–3 years of life, delayed diagnoses may result in cases manifesting at a later stage (7). In Palestine, numerous patients may remain undetected owing to a lack of awareness of the disease. Consequently, a significant educational contribution of this study is to increase knowledge among Palestinian physicians, prompting them to consider CGD in patients presenting during late infancy. This study revealed that 11 of the 14 CGD patients were diagnosed within the past five years. These findings indicate that medical improvements are enabling earlier diagnosis, providing optimism for improved detection and management of the disease in the future. To the best of our knowledge, this study of CGD is the first in Palestine.

## 5 Conclusion

This study identified 14 Palestinian patients with CGD who presented at a young age (median age: 8.5 months), although some patients had late-onset disease (up to 14 years). The patients displayed various clinical features, with lymphadenopathy (71.4%) and chronic diarrhea (64.2%) being the most prevalent, highlighting that this condition can be encountered by physicians in diverse clinical settings. There were no significant or medically managed reactions to the BCG vaccine. The genetic variants observed in our patients were diverse, likely influenced by the significant prevalence of consanguineous marriages within the study group (57.14%); most cases exhibited an AR inheritance pattern (78.6%), with an estimated mortality rate of 14.28% (2 out of 14 patients).

In conclusion, this study identified several key features among Palestinian CGD patients who could assist physicians in suspecting the disease. Early detection facilitates timely intervention, markedly enhancing patients’ quality of life and reducing the risk of life-threatening complications.

## 6 Acknowledge study limitations

The present study has numerous limitations that must be considered. The limited sample size (n=14) may restrict the generalizability of the findings, particularly for the male-to-female ratio, which deviates from the global norm. This small sample size is primarily due to the extreme rarity of CGD, coupled with a likely history of underdiagnosis caused by low medical awareness of the condition in Palestine. The study’s context posed significant challenges; owing to the absence of local facilities for genetic testing, materials had to be dispatched internationally, resulting in significant delays in diagnosis. Furthermore, the widespread use of broad-spectrum antibiotics prior to hospital admission likely influences microbiological cultures, potentially leading to an underestimation of causative agents. The limited frequency of hematopoietic stem cell transplantation (HSCT) is influenced by logistical challenges, including the difficulty in locating compatible donors and the inability to perform treatment locally. The retrospective nature of the data and the potential unawareness of symptoms by caregivers may have influenced the precise documentation of adverse events, particularly those related to BCG vaccination. These variables underscore the need for future research with larger sample sizes and improved diagnostic capabilities.
