# Correction: First review of chronic granulomatous disease in Palestine: clinical and genetic characteristics

**DOI:** 10.3389/fimmu.2026.1865717

**Published:** 2026-05-06

**Authors:** Fatima az-Zahra Thawabteh, Sedrah Abu Rmilah, Aya Siaj, Rawan Abu khdair, Rabee Adwan

**Affiliations:** 1 Medical Research Club, Faculty of Medicine, Al-Quds University, Jerusalem, Palestine; 2 Infectious Diseases Specialist, Al-Makassed Hospital, East-Jerusalem, Palestine; 3 Pediatrics Department, Faculty of Medicine, Al-Quds University, Jerusalem, Palestine

**Keywords:** Chronic granulomatous disease, clinical characteristics, genetics, immunodeficiency, lymphadenopathy, Palestine

The Affiliation “Pediatrics Department, Faculty of Medicine, Al-Quds University, Jerusalem, Palestine” was omitted for author “Rabee Adwan”. This has now been added for author “Rabee Adwan”.

The original version of this article has been updated.

